# Extrafollicular Plasmablasts Present in the Acute Phase of Infections Express High Levels of PD-L1 and Are Able to Limit T Cell Response

**DOI:** 10.3389/fimmu.2022.828734

**Published:** 2022-05-16

**Authors:** Melisa Gorosito Serrán, Facundo Fiocca Vernengo, Laura Almada, Cristian G. Beccaria, Yamila Gazzoni, Pablo F. Canete, Jonathan A. Roco, Jimena Tosello Boari, Maria Cecilia Ramello, Ellen Wehrens, Yeping Cai, Elina I. Zuniga, Carolina L. Montes, Ian A. Cockburn, Eva V. Acosta Rodriguez, Carola G. Vinuesa, Adriana Gruppi

**Affiliations:** ^1^ Centro de Investigaciones en Bioquímica Clínica e Inmunología (CIBICI)-Consejo Nacional de Investigaciones Científicas y Técnicas (CONICET), Facultad de Ciencias Químicas, Universidad Nacional de Córdoba, Córdoba, Argentina; ^2^ Department of Immunology and Infectious Disease, John Curtin School of Medical Research, Australian National University, Canberra, ACT, Australia; ^3^ Division of Biological Sciences, University of California, San Diego, San Diego, CA, United States; ^4^ China-Australia Centre for Personalised Immunology, Shanghai Renji Hospital, Shanghai Jiao Tong University, Shanghai, China

**Keywords:** plasmablasts/plasma cells, B cell, PD-L1, *Trypanosoma cruzi*, *Plasmodium*, LCMV, immunosuppression, microorganism evasion

## Abstract

During infections with protozoan parasites or some viruses, T cell immunosuppression is generated simultaneously with a high B cell activation. It has been described that, as well as producing antibodies, plasmablasts, the differentiation product of activated B cells, can condition the development of protective immunity in infections. Here, we show that, in *T. cruzi* infection, all the plasmablasts detected during the acute phase of the infection had higher surface expression of PD-L1 than other mononuclear cells. PD-L1^hi^ plasmablasts were induced *in vivo* in a BCR-specific manner and required help from Bcl-6^+^CD4^+^T cells. PD-L1^hi^ expression was not a characteristic of all antibody-secreting cells since plasma cells found during the chronic phase of infection expressed PD-L1 but at lower levels. PD-L1^hi^ plasmablasts were also present in mice infected with *Plasmodium* or with lymphocytic choriomeningitis virus, but not in mice with autoimmune disorders or immunized with T cell-dependent antigens. *In vitro* experiments showed that PD-L1^hi^ plasmablasts suppressed the T cell response, partially *via* PD-L1. Thus, this study reveals that extrafollicular PD-L1^hi^ plasmablasts, whose peaks of response precede the peak of germinal center response, may have a modulatory function in infections, thus influencing T cell response.

## Highlights

Pathogens develop different strategies to settle in the host. We identified a plasmablast population that expressed high levels of PD-L1, induced by pathogens in acute infections which influences T cell response.

## Introduction

Persistent replicating parasites and viruses can overcome the initial immune response by suppressing the innate and T cell response to allow the establishment of chronic infection. Immune suppression in chronic infections serves to prevent pathogenic immune-mediated inflammation and to suppress protective effector immune responses (1, 2). As such, *Trypanosoma cruzi* infection, also known as Chagas disease, has been an important model for understanding the delicate balance between protective T cell immunity, immune suppression, and parasite establishment.

Chagas disease presents an acute phase both in mice and humans, characterized by a state of immunosuppression in which *T. cruzi* replicates extensively and induces immunomodulatory molecules that delay parasite-specific T cell effector responses (3–6). It has been reported that during this phase of *T. cruzi* infection the expression of PD-1, one of the members of the CD28/CTLA4 family with inhibitory capacity, increases on the myocardium infiltrating CD4^+^ and CD8^+^ T cells (7). The cross-linking of PD-1 with any of its two ligands, PD-L1 (8) or PD-L2 (9), inhibits the activation of T cells and the production of IL-2 and IFNγ.

Many studies have shown that other protozoan parasites, such as *Plasmodium* (10) as well as viruses (11) and bacteria (12), also benefit from the PD-1/PD-L1 pathway to suppress and evade the host’s adaptive immunity. The activation of the PD-1/PD-L1 pathway might enable the establishment of microorganisms and chronicity (13–15). Accordingly, the blocking of PD-1/PD-L1 signals, in combination with therapeutic immunizations or therapies with cytokines, has been proposed as a strategy to improve the efficacy of vaccinations (16) and to revitalize exhausted T cells (17).

During infection with *T. cruzi*, blocking PD-1 and PD-L1 or deletion of the PD-1 gene result in a reduction in parasitemia and tissue parasitism, but also in increased mortality due to an augmented cardiac inflammatory response (7). These results reveal that the signaling pathway of PD-1/PD-L1 has an important role in the control of inflammation induced by *T. cruzi* and provide another perspective on the mechanisms of regulation in the pathogenesis of Chagas disease. However, PD-L1-expressing regulatory cells have not been fully identified in this context.

It has been reported that PD-L1^+^ regulatory plasmablasts have a critical role in suppressing immune T cell responses in cancer models (18); however, their role in chronic infection has not been described. Recently, we reported that Blimp-1^fl/fl^CD23^Cre^ mice, which are deficient in plasmablasts, had a significantly higher number of trypomastigotes in blood and splenic TNF^+^CD4^+^ T cells than infected CD23^Cre^ mice after *T. cruzi* infection (19), suggesting that plasmablasts and plasma cells are important for parasite replication control and also for the regulation of TNF-producing cells. Considering that the absence of plasmablasts improves effector T cell responses, we explored whether PD-L1^+^ plasmablasts are present in *T. cruzi*-infected mice and in other parasitic and chronic infections. Moreover, given the proximity of extrafollicular plasmablasts to T cell zones (20), we asked whether PD-L1^+^ plasmablasts can regulate the T cell response *via* the PD-1/PD-L1 pathway.

Here we show that, in *T. cruzi* infection, all the plasmablasts detected had higher surface expression of PD-L1 than other B-lymphocyte lineage cells or other mononuclear cells. These plasmablasts were also present in other infections which involve high inflammatory conditions. This study reveals that extrafollicular PD-L1^hi^ plasmablasts *in vitro* can operate as suppressor cells on PD-1 expressing T cells, providing new insights that may be harnessed for cell targeting immune therapies.

## Results

### The Plasmablast Response Peaks Before the Peak of the GC Reaction and Declines Before CD4+ T Cells Peak

**Figure 1:**
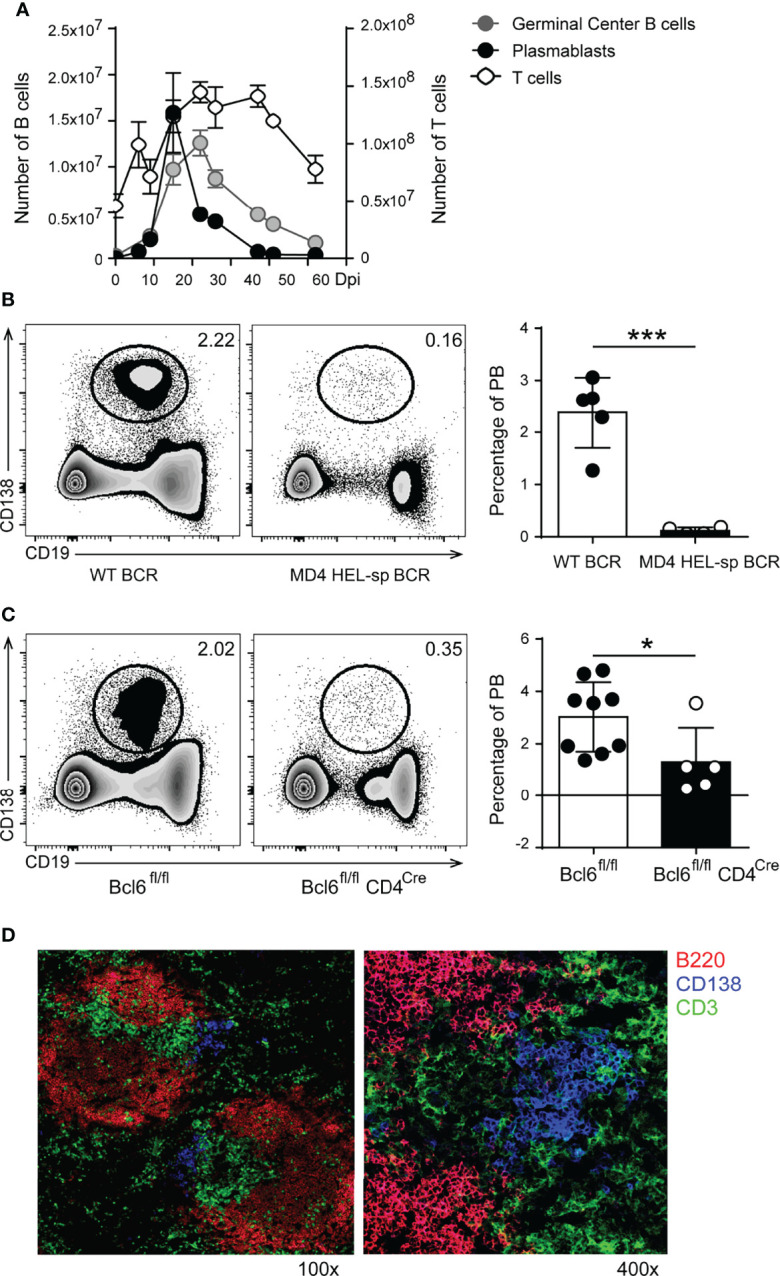
In T. cruzi infection, the plasmablast response peaks before GC reaction, was generated in a BCR specific manner and required Bcl-6^+^CD4^+^T cell collaboration. **(A)** Kinetics of plasmablasts (PB, CD19^int^B220^low^CD138^hi^), Germinal Center (GC) B cells (CD19^+^CD3^-^CD138^-^ B220^+^CD38^low^Bcl6^+)^ and CD3^+^CD4^+^ T cells number in the spleen of T. cruzi infected mice. **(B)** Representative flow cytometry plots and statistical analysis of the percentage of splenic PB (CD138^+^CD19^int^/^low^) from WT (WT BCR, n = 5) and MD4 (HEL-sp BCR, n = 8) mice at 14 Dpi with T. cruzi (***p < 0.001, two tailed t Test). **(C)** Representative flow cytometry plots and statistical analysis of the percentage of splenic PB from Cre negative littermates Bcl6^fl/fl^ (n = 9) and Bcl6^fl/fl^CD4^Cre^ (Bcl-6^neg^CD4+ T cells, n = 5) mice at 14 Dpi with T. cruzi. (*p < 0.05, two tailed t Test) **(D)** Representative Immunofluorescence of spleen sections (7um) from T. cruzi-infected C57BL/6 mice obtained at 14 Dpi, stained with anti-B220 (red), anti-CD3 (green) and anti-CD138 (blue). Magnification: x100 (left) and x400 (right) (n = 9). Results shown in **(A, D)** are representative of five independent experiments. Data in **(B, C)** are representative of two independent experiments.

To understand the mechanisms by which plasmablasts regulate the CD4^+^ T cell response in experimental Chagas disease, we first investigated the kinetics of the plasmablast response. Compared with uninfected mice, flow cytometry analysis revealed that the number of splenic plasmablasts (CD3^-^B220^low^IgD^-^CD138^+^) increased prior to the GC reaction and peaked at 15-18 days post infection (Dpi) with *T. cruzi* (**Figure 1A**). The number of plasmablasts declined at 20 Dpi, whereas the number of GC B cells (CD19^+^CD3^-^CD138^-^B220^+^CD38^low^Bcl6^+^) remained high until 25 Dpi and decreased later. The peak of the total CD4^+^ T cell response occurred after the frequency and number of plasmablasts had decreased (**Figure 1A**). This cellular kinetics may be related to the pathogen load since the parasitemia peaks at 10-12 Dpi (19).

### Plasmablasts Are Generated in an Antigen-Specific and Tfh-Dependent Manner

To understand in more depth the origin and characteristics of plasmablasts in *T. cruzi* infection, we infected MD4 mice (whose B cells express a transgenic BCRs specific for a *T. cruzi* non-related protein, HEL) and their non-transgenic controls (WT-BCR, whose B cells express a wild-type BCR repertoire). **Figure 1B** illustrates a representative staining of CD19 and CD138 on lymphocytes from the spleen of *T. cruzi*-infected WT-BCR and MD4 mice. Infection of MD4 mice showed that plasmablast induction depends on BCR signaling, since plasmablasts were almost absent from infected MD4 mice compared to WT mice around the peak of the plasmablast responses at 14 Dpi (**Figure 1B**, left and right graphs).

Since extrafollicular (EF) antibody responses require Bcl-6 expression by T cells (21), we investigated whether plasmablast induction and/or survival required the collaboration of Bcl-6^+^CD4^+^T cells. For this, we took advantage of Bcl6^fl/fl^CD4^Cre^ mice that lack Tfh and CXCR5^neg^Bcl-6^+^CD4^+^ T cells. The dot plots (**Figure 1C**) show representative data illustrating the identification of CD19^int/low^CD138^+^ cells among lymphocytes from the spleen of *T. cruzi* Bcl6^fl/fl^ and Bcl6^fl/fl^CD4^Cre^ infected mice. While Cre-negative littermates develop a normal Bcl-6^+^CD4^+^T cell response (22), *T. cruzi*-infected Bcl6^fl/fl^CD4^Cre^ mice had a significant decrease in the frequency of plasmablasts (**Figure 1C**, left and right graph), indicating that the plasmablast response is critically dependent on Bcl-6^+^CD4^+^ T cells.

Immunofluorescence analysis of the spleen of *T. cruzi*-infected mice (**Figure 1D**) identified CD138^+^ cells extending beyond the classical sites of extrafollicular plasmablast growth (**Figure 1D**, CD138^+^ cells, blue) into the T zone areas (**Figure 1D**, CD3^+^ cells, green), a finding consistent with our previous reports (20, 23). The results suggest that, in *T. cruzi* infection, plasmablasts were induced in a BCR-dependent manner, required Bcl-6^+^CD4^+^ T cell collaboration, and were located at extrafollicular sites in close proximity to T cells.

### Plasmablasts From *T. cruzi*-Infected Mice Express High Levels of PD-L1

**Figure 2:**
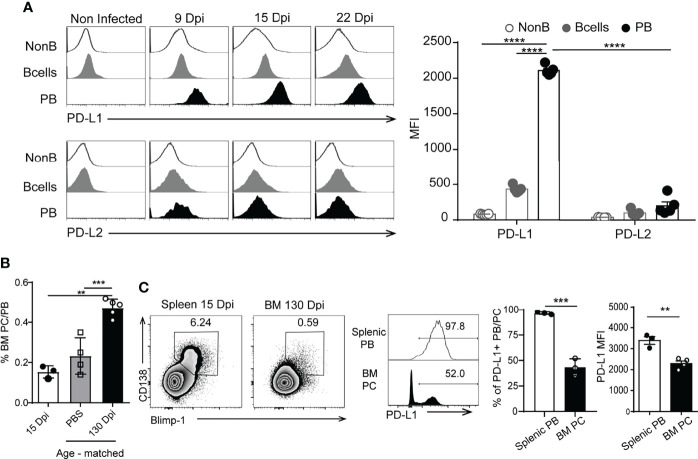
Plasmablasts from *T. cruzi*-infected mice expressed high levels of PD-L1.** (A)** Representative histograms of PD-L1 or PD-L2 expression on splenic non-B cells (CD19^-^CD138^-^B220^-^), B cells (CD19^+^CD3^-^CD138^-^B220^+^) from non-infected mice (n=5) and *T. cruzi*-infected C57BL/6 mice (n=5) and on plasmablasts (PB) (CD19^int^B220^low^CD138^hi^) from *T. cruzi*-infected C57BL/6 mice evaluated at 9, 15 and 22 Dpi. Statistical analysis of mean fluorescence intensity (MFI) of PD-L1 or PD-L2 on the mentioned cells analyzed at 15 Dpi. ****p < 0.0001 one-way ANOVA, Bonferroni *post hoc* test. **(B)** Statistical analysis of the frequency of PB/PC (plasma cell) CD138^+^Blimp-1^+^ of live single lymphocytes (gated on IgD^-^IgM^-^CD11b^-^CD24^-^) from bone marrow (BM) obtained at 15 Dpi (white bar) and at 130 Dpi with *T. cruzi* (black bar). Cells from BM from age-matched non-infected mice, injected with PBS, were used as controls (gray bar). **(C)** Representative plots of CD138 vs Blimp-1 of live single lymphocytes (gated on IgD^-^IgM^-^CD11b^-^CD24^-^) from spleen and bone marrow (BM) obtained at 15 Dpi and 130 Dpi with *T. cruzi*, respectively. Representative histograms of PD-L1 on gated CD138^+^Blimp-1^+^B220^+^ cells. Numbers in histograms indicate the percentage of PD-L1^+^ cells. Bar graph shows statistical analysis of the frequency of PD-L1^+^ PB/PC and MFI of PD-L1^+^ on splenic PB from 15 Dpi-Tcruzi infected mice (white bar) and 130 Dpi-BM plasma cells (PC). **p < 0.01, one-way ANOVA, Bonferroni *post hoc* test. Data in **(A, B)** are representative of three independent experiments. ***p < 0.001.

To identify if plasmablasts express inhibitory molecules able to modulate the T cell response, we evaluated the expression of PD-L1 and PD-L2 (24) on plasmablasts from *T. cruzi*-infected mice. In comparison with B and non-B cells, plasmablasts from *T. cruzi*-infected mice, evaluated at different Dpi, expressed the highest levels of PD-L1 (determined as MFI, **Figure 2A**). Most of the plasmablasts (nearly 97% of them) detected during the acute phase of *T. cruzi* infection expressed high levels of PD-L1 (**Figures 2A, B**). As a consequence of the infection, B cells and plasmablasts also expressed PD-L2, but at similar levels (**Figure 2A**, see histograms and bar graph). PD-L1^hi^ plasmablasts were absent in the spleen of infected mice during the chronic phase of the infection (130 Dpi, data not shown). The frequency of CD138^+^ Blimp-1^+^ cells in the spleen and bone marrow of infected mice, obtained at 15 and 130 Dpi, is illustrated in the dot plots of **Figure 2B**. As a control, the frequency of CD138^+^ Blimp-1^+^ cells in the bone marrow of age-matched non-infected mice is also shown (**Figure 2B**). CD138^+^ Blimp-1^+^ cells in the bone marrow of 15 Dpi-infected mice were few, and the frequency of these cells was significantly higher in the bone marrow of 130 Dpi-infected mice than in age-matched non-infected mice or 15 Dpi-infected mice (**Figure 2B**). While most plasmablasts analyzed during the acute phase were PD-L1^+^ (**Figure 2C**, grey histogram), only ~ 50% of the bone marrow plasma cells detected in the chronic phase (130 Dpi) expressed PD-L1 (**Figure 2C**, black histograms). PD-L1 expression on plasma cells detected in chronic infection was significantly lower (**Figure 2C**, bar graph, black bar) than PD-L1 expression on plasmablasts present at 15 Dpi (**Figure 2C**, bar graph, white bar).

### PD-L1^hi^ Plasmablasts Are Also Detected in Other Infections But Not in Autoimmune or in Immunized Mice

**Figure 3:**
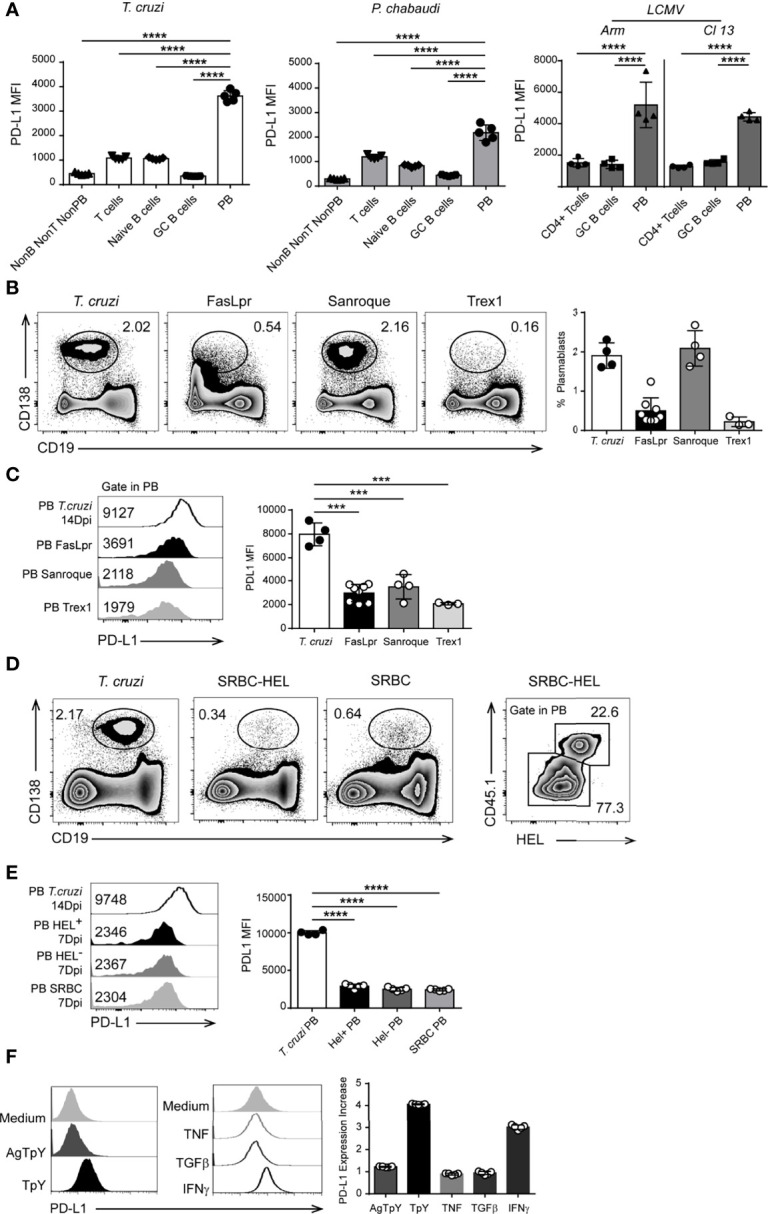
PD-L1^hi^ plasmablasts were present in infected but not in autoimmune mice. **(A)** Statistical analysis of MFI of PD-L1 on Non-B non-T non-PB cells (CD3^-^CD19^-^B220^-^CD138^-^), T cells (CD3^+^CD19^-^B220^-^), naïve B cells (CD19^+^CD3^-^CD138^-^B220^+^IgD^+^CD38^+^Bcl6^-^), GC B cells (CD19^+^CD3^-^CD138^-^B220^+^CD38^low^Bcl6^+^) and plasmablasts (PB, CD19 ^int^CD138^+^) from the spleen of *T. cruzi-*infected mice (n=5) obtained at 14 Dpi, or from the spleen of *P. chabaudi*-infected mice obtained at 10 Dpi (n=5); or CD4+ T cells, GC B cells, and PB from the spleen of Arm- or Cl13 LCMV-infected mice obtained at 9 Dpi (n = 4 for each LCMV infection). ****p < 0.0001, one-way ANOVA, Bonferroni *post hoc* test. **(B)** Representative flow cytometry plots and statistical analysis of the percentage of splenic plasmablasts from *T. cruzi-infected-* and from *FasLpr, Sanroque* and Trex1 mice. C57BL6 mice (n = 4) were infected with *T. cruzi* Y strain, and the spleens were obtained at 14 Dpi. Spleens from *FasLpr (n = 7), Sanroque (n = 4)* and Trex1 *(n = 3)* mice were obtained at 10-12 weeks of age. Splenocytes were stained with anti-B220, anti-CD138 and anti-PD-L1. **(C)** Representative histograms and statistical analysis of PD-L1 expression (MFI) on plasmablasts (PB) from the spleen of *T cruzi-*infected mice and FasLpr, Sanroque and Trex1 mice. **(D)** Representative flow cytometry plots of the percentage of plasmablasts from 14 Dpi-*T. cruzi*-infected mice and from mice immunized with 2x10^8^ SRBC or with SRBC conjugated with HEL. SW_HEL_-specific B cells identified as CD45.1^+^HEL^+^ cells and the percentage of SW_HEL_-specific plasmablasts CD138^+^CD19^low^ in CD45.2 mice transferred with 30.000 HEL-specific B cells and immunized with 2x10^8^ SRBC conjugated with HEL. **(E)** Representative flow cytometry histograms and statistical analysis of PD-L1 expression (MFI) on plasmablasts from *T. cruzi*-infected mice (n = 4), SW_HEL_-specific (PB HEL^+^) and -non-specific (PB HEL^-^) plasmablasts from CD45.2 mice transferred with 30,000 HEL-specific B cells and immunized with 2x10^8^ SRBC conjugated with HEL (n = 5), and on plasmablasts from mice immunized with SRBC (7 DPI) (PB SRBC, n = 5). **(F)** Representative histograms of PD-L1 expression on B cells cultured with medium, *T. cruzi* trypomastigote antigens (AgTpY), live *T. cruzi* trypomastigotes (TpY), TNF, TGFβ, and IFNγ. Bar graph shows statistical analysis of the PD-L1 expression increase, described as a fold change, on B cells cultured with the mentioned stimulus respect to medium (n = 5). ***p < 0.001, ****p < 0.0001 one-way ANOVA, Bonferroni *post hoc* test. Data in **(A)** are representative of three independent experiments and in **B–F**) are representative of two independent experiments.

We also investigated whether PD-L1^hi^ plasmablasts are unique to *T. cruzi*-infected mice or if they are present in other persistent infections. We also analyzed the expression of PD-L1 on other B lineage cells. We evaluated the presence of PD-L1^hi^ plasmablasts in mice infected with the Armstrong strain of LCMV, which causes acute infection, or with the LCMV clone 13, which causes chronic infection (25). Analysis of the spleens of mice infected with LCMV (either Armstrong –ARM- or clone 13 –Cl13- isolates) or with *P. chabaudi* analyzed after 9 and 10 Dpi, respectively, showed similar frequencies of PD-L1^+^plasmablasts (**Figure S1**), and that almost all the plasmablasts were PD-L1^+^ (**Figure S1** and **Figure 3A**). In a similar way to our findings with *T. cruzi*, the plasmablasts from *P. chabaudi* and from ARM- or Cl13-LCMV-infected mice also expressed high levels of PD-L1 in comparison to naive and GC B cells and T cells from the same mice (**Figure 3A**).

We next investigated whether PD-L1^hi^ plasmablasts occur in autoimmune disease or after immunization with non-microbial protein antigens. Mice known to develop spontaneous autoimmunity, such as *Fas^lpr^*, *Sanroque* and *Trex1*-deficient mice, had varying frequencies of splenic plasmablasts (**Figure 3B**, representative dot plots and bar graphs). Regardless of the frequency of plasmablasts present in the autoimmune mice, PD-L1 expression on splenic plasmablasts determined as MFI from autoimmune mice was significantly lower than on plasmablasts in mice infected with *T. cruzi* (**Figure 3C**, representative histograms and bar graphs), although they remained positive compared to the isotype control.

To test whether the protein antigen-immunization that induces EF plasmablasts triggers PD-L1 expression on plasmablasts, we used the SwHEL cell transfer model (26). HEL-specific B cells from SwHEL mice (CD45.1^+^, which contain a large population of B cells with BCR that recognize HEL, hen egg lysozyme) were adoptively transferred into C57BL/6 mice, which were then immunized iv with sheep red blood cells (SRBC) alone (immunization control) or SRBC conjugated to HEL2x (26). PD-L1 expression was analyzed on CD19^low^CD138^+^ cells gated in CD45.1^+^HEL^+^ cells (**Figure 3D**). PD-L1 expression at the peak of the EF HEL-specific plasmablast response (day 6) was significantly lower than that from plasmablasts at the peak of acute *T. cruzi* infection (14 Dpi) (**Figure 3E**, histograms - PB *T cruzi* MFI: 9748 vs PB HEL^+^ MFI: 2346). Importantly, HEL-specific plasmablasts had a PD-L1 expression similar to that of non-HEL binding plasmablasts and that of plasmablasts generated by SRBC alone (**Figure 3E**). Overall, the results suggest that BCR binding on B cells was not sufficient to induce high PD-L1 expression on plasmablasts.

Since PD-L1^hi^ expression on plasmablasts was observed in infected mice, we hypothesize that a strong inflammatory environment or the pathogens favor the high expression of PD-L1 on plasmablasts. To test this, purified B cells from uninfected C57BL/6 mice were cultured in the presence or absence of *T. cruzi* antigens, live trypomastigotes, or cytokines, and PD-L1 expression was evaluated after 24 h. IFNγ, TNF and TGFβ were the selected cytokines and these were tested as PD-L1-inducers on B cells because they have been shown to increase PD-L1 expression on other cell types (27–29), and they are present at high levels during the acute phase of *T. cruzi* infection, when PD-L^hi^ plasmablasts are present (19, 30).

In comparison to B cells cultured with medium alone, PD-L1 upregulation was observed when B cells were cultured with live trypomastigotes or with recombinant IFNγ but not with *T. cruzi* antigens, TNF, or TGFβ (**Figure 3F**). However, PD-L1^hi^ plasmablasts were present in IFNγ-/- mice infected with *T. cruzi* (**Figure S2**), indicating that this cytokine may be redundant with other cytokines for mediating PD-L1 upregulation on plasmablasts *in vivo*. Together, these results suggest that live parasites are required to induce maximal PD-L1 expression, possibly through inducing a combination of pro-inflammatory cytokines.

### Plasmablasts Regulate IFNγ- and TNF-Producing Cells *via* PD-L1

**Figure 4:**
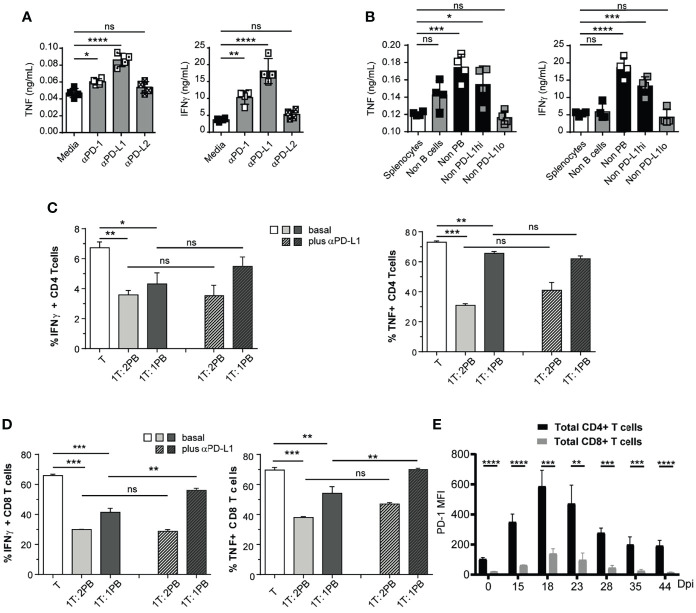
Plasmablasts from *T. cruzi*-infected mice suppressed CD8+ T cell response *via* PD-L1. **(A)** 1 x 10^5^ Splenocytes from *T. cruzi*-infected mice obtained at 14 Dpi were incubated with *T. cruzi* antigens and cultured for 48h with media, anti-PD-1, anti-PD-L1, or anti-PD-L2. TNF and IFNγ concentrations were determined by Elisa in the culture supernatant. **(B)** 1x 10^5^ Non-B (CD19^neg^CD138^neg^) cells in a suspension of total splenocytes (non-depleted) from *T. cruzi* infected mice obtained at 14 Dpi or depleted of B cells (Non-B cells), plasmablasts (Non-PB), PD-L1^hi^ cells (Non-PD-L1^hi^) or PD-L1^low^ (Non-PD-L1^lo^) cells were cultured with *T. cruzi* antigens for 48h. TNF and IFNγ concentrations were determined by Elisa in the culture supernatant. **(C)** Purified CD4^+^ and **(D)** CD8^+^ **(**responder T cell from non-infected mice activated with anti-CD3 plus anti-CD28 were co-cultured with sorted plasmablast from *T. cruzi*-infected mice obtained at 14 Dpi in 1:1 or 1:2 ratio with for 3 days, in the presence of control antibody (basal) or anti-PD-L1. Bars show the frequency of TNF^+^ and IFNγ^+^ CD4^+^ or CD8^+^ T cells which were determined by flow cytometry. **(E)** PD-1 expression, determined as MFI by flow cytometry, of splenic CD4^+^ (black) and CD8^+^ (grey) T cells obtained from *T. cruzi*-infected mice at different Dpi. Data in **(A–D)** are representative of two independent experiments. ta are shown as mean ± SD of triplicates cultures. *p < 0.05, **p < 0.01, ***p < 0.001, ****p < 0.0001 two-tailed t Test). ns, non significant.

To test the role of PD-L1 on TNF- and IFNγ-producing cells from *T. cruzi-*infected mice, splenocytes stimulated with *T. cruzi* antigens were cultured with medium or with anti-PD-1, anti-PD-L1, or anti-PD-L2 and media, with isotypes as controls, and the concentration of cytokines was evaluated in the culture supernatant. The blockade of PD-1 and PD-L1 signaling significantly increased TNF and IFNγ concentration in the culture supernatants (**Figure 4A**). As expected, no significant changes were detected in the concentration of cytokines in the supernatant of splenocytes cultured with anti-PD-L2 compared to splenocytes cultured with medium (**Figure 4A**), indicating, as expected, that the PD-L2 pathway was not involved in the *in vitro* regulation of cytokine-producing splenic cells from *T. cruzi-*infected mice.

To identify the B-lineage cell population responsible for limiting cytokine-producing T cells, splenocytes from *T. cruzi*-infected mice obtained at 14 Dpi were depleted of plasmablasts or B cells by cell sorting (**Figure S3A–C**). Later, the cells were cultured with parasite antigens, and cytokine concentration was determined in culture supernatants. Only plasmablast depletion significantly increased the concentration of TNF and IFNγ in the culture supernatant (**Figure 4B**). Depletion of PD-L1^hi^ cells, but not of PD-L1^low^ cells, from splenocytes also led to an increase in the concentration of cytokines in the supernatant (**Figure 4B**). Given that depletion of PD-L1^hi^ cells concomitantly leads to plasmablast elimination (**Figure S3D, E**), these data suggest that plasmablasts with high PD-L1 expression inhibit cytokine-producing cells *in vitro*.

Next, we investigated the regulatory capacity of PD-L1^hi^ plasmablasts on CD4^+^ and CD8^+^ T cell cytokine production. For this, sorted plasmablast from 14 Dpi-*T. cruzi*-infected mice were incubated with purified splenic CD4^+^ or CD8^+^ T cells obtained from the spleen of non-infected control mice in a plate coated with anti-CD3/CD28. Three days after co-culture, the intracellular cytokine content of T cells was assessed by FACS. When T cells were co-cultured with plasmablasts, the frequency of TNF^+^ and IFNγ^+^ CD4^+^ or CD8^+^ T cells significantly decreased (**Figures 4C, D**, plain light and dark gray bars).

To explore the role of PD-L1, we performed blocking experiments by adding an anti-PD-L1 mAb from the beginning of the co-culture. PD-L1 blockade increased the frequency of IFNγ- and TNF-producing CD8^+^ T cells. In contrast to CD8^+^ T cells, the effect of anti-PD-L1 blockade in co-culture with CD4^+^ T cells was less pronounced or absent (**Figure 4C**, plain gray bars vs striped gray bars). Next, we evaluated the expression of PD-1, the target of PD-L1, on T cells from *T. cruzi-*infected mice at different times post infection. **Figure 4E** shows PD-1 expression of splenic CD4^+^ and CD8^+^ T cells, determined as MFI by flow cytometry, obtained at different Dpi. We observed that splenic CD4^+^ T cells from *T. cruzi*-infected mice had significantly higher expression of PD-1 than CD8^+^ T cells. Thus, regardless of PD-1 expression, CD8^+^ T cells were more susceptible to PD-L1 expressing plasmablast suppression.

## Discussion

Our current understanding of humoral responses has been derived largely from immunization with model antigens. In the last decade, murine models of infections that also present a significant inflammatory component have enabled new cell populations to be identified with fundamental roles in the induction of the B cell response (31, 32), the discovery of novel sites where B cells are activated (33) and the identification of distinct regulatory B cell functions (23, 34). Here, we describe plasmablasts with a very high expression of PD-L1 that were detected in infected mice but were absent in mice with autoimmunity or immunized with T-dependent model antigens. Our findings provide the first evidence that most of the plasmablasts in *T. cruzi*, *Plasmodium* or LCMV infections express high levels of PD-L1, in comparison to naïve and GC B cells, T cells or other lymphoid cells. Interestingly, CD138^+^B220^low^Blimp-1^hi^ cells present in bone marrow at the chronic phase of *T. cruzi* infection did not exhibit high expression of PD-L1, suggesting that PD-L1^hi^ expression is not a characteristic of all antibody-secreting cells. PD-L1 expression was highest in one antibody-secreting cell population, the plasmablasts, present in the early acute phase of the infection, when there is a strong inflammation, and the effector response of T cells begins to develop (35).

Most of the plasmablasts detected during the acute phase of *T. cruzi* infection are PD-L1^hi^ and are extrafollicular since the CD138^+^B220^low^ cells, located outside the follicle, are the only antibody-secreting cells expressing CD138 at this time of infection (20) and **Figure 1**). The peak of PD-L1^hi^ plasmablasts precedes the peak of GC reaction and, as previously reported for the extrafollicular response (21), Bcl-6 expression in T cells is essential for their optimal induction/survival. Consistent with this, very few PD-L1^hi^B220^low^ cells were detected in the spleens of infected mice lacking Bcl-6 expression in CD4^+^T cells. Additionally, despite the prevailing view that, during the acute phase of *T. cruzi* infection most B cells lack parasite specificity, suggesting non-antigen specific B cell activation (36), PD-L1^hi^ plasmablasts required parasite-specific BCRs. Indeed, mice whose B cells expressed BCRs non-specific for *T. cruzi* exhibited a very low number of plasmablasts at 14 Dpi.

High PD-L1 expression on antibody-secreting cells was previously reported on intestinal lamina propria IgA^+^ plasma cells, which are the major PD-L1 expressing cells in that tissue. PD-L1 expression levels on intestinal IgA plasma cells are higher than those on IgG plasma cells in peripheral lymphoid tissues (37). Probably, the high expression of PD-L1 in intestinal IgA plasma cells is triggered by microbiota, as we observed that microorganisms (in this report, *T. cruzi*) *per se* and/or inflammatory cytokines can induce high PD-L1 expression on B cells. PD-L1 expression on plasmablasts was also described in different mouse prostate cancer models under Oxaliplatin treatment (18). This therapy induces tumor-infiltrating plasmacytes that express IgA, IL-10 and PD-L1. Elimination of these cells, which also infiltrate human-therapy-resistant prostate cancer, allows cytotoxic T cell-dependent eradication of Oxaliplatin-treated tumors (18), suggesting a suppressive function in this population. Considering that PD-L1 plays a major role in suppressing the adaptive arm of the immune system, PD-L1^hi^ plasmablasts emerge as a population with regulatory capacity.

The majority of studies on Breg cells have focused on the predisposition of immature and mature B cells to produce IL-10 and to suppress a range of autoimmune or allergic conditions in both mice and humans (38). Little information is available about the regulatory function and mechanisms of action of antibody-secreting cells. In this study, we found that plasmablasts from *T. cruzi*-infected mice exert a regulatory function through PD-L1 *in vitro* on T cells. When splenocytes from *T. cruzi*-infected mice were depleted from PD-L1^hi^ cells or plasmablasts and later cultured with *T. cruzi* antigens, we observed a greater increase in TNF and IFNγ levels in the culture supernatant than in the culture of total splenocytes. We observed that *T. cruzi* plasmablasts suppressed both the CD4^+^ and the CD8^+^ T cell response. Analyzing possible targets of PD-L1^hi^ plasmablasts, we identified CD8^+^ T cells as targets of PD-L1–expressing plasmablasts. It should be noted that, although there was a tendency of anti-PD-L1 to revert the suppression of CD4^+^ T cells triggered by plasmablasts, this was not significant, suggesting that plasmablasts may suppress the CD4^+^ T cell response using other mechanisms.

The immunomodulatory effect exerted by plasmablasts may involve more than one mediator. It has been described that IL-10-producing plasmablasts exert regulatory functions in autoimmunity (39) and we observed that plasmablasts from *T. cruzi*-infected mice produce IL-10 (data not shown). Additionally, it was reported that a high consumption of glutamine by plasmablasts can deprive other populations of substrate, conditioning their response (40). Whether these mechanisms operate in *T. cruzi* infection needs to be determined.

Consistent with our previous report on B cells from patients with rheumatoid arthritis (41), this study reveals that extrafollicular PD-L1^hi^ plasmablasts, which precede the GC response, can operate *in vitro* as modulatory cells on PD-1 expressing CD8^+^ T cells, providing new insights that could be harnessed for cell targeting immune therapies. It has been described that human and mouse glioblastoma-associated B cells that overexpress PD-L1 also have immunosuppressive activity directed towards activated CD8^+^ T cells (42). B cells located in the tumor microenvironment, but not circulating or from lymph nodes, express high levels of PD-L1, but this expression apparently does not depend on the inflammatory environment but rather tumor myeloid-derived suppressive cells deliver microvesicles transporting PD-L1, which is taken up by tumoral B cells.

Plasmablasts also express PD-L2, another molecule that interacts with PD-1 and suppresses T cell proliferation and cytokine release (9). However, we observed that PD-L2 was not mediating effector cell suppression, since PD-L2 blockade in cell culture did not affect cytokine production.

The data obtained in this work allow us to partially explain why, during infections with protozoan parasites such as *T. cruzi* and *Plasmodium*, a delay is generated in T cell response simultaneously with high B cell activation. The acute phase of Chagas disease in mice and humans is marked by a state of immunosuppression, which coexists with polyclonal B cell activation, in which *T. cruzi* replicates extensively and induces immunomodulatory molecules that delay parasite-specific responses mediated by effector T cells (43). Also, it has been reported that, in the acute phase of infection with *P. chabaudi*, the composition of the B cell compartment is altered, with vigorous extrafollicular growth of plasmablasts and GC formation. In *Plasmodium* infection, extrafollicular foci of plasmablasts are visible from day 4, initiating a very strong plasma cell response. By day 10, plasma cells are localized in the periarteriolar region of the white pulp and form clusters, occupying part of the area normally filled by T cells. During this phase of infection, as in *T. cruzi* infection, there is a delayed GC response and a significant reduction of the humoral response to T-dependent antigens (44).

Finally, we observed that mice infected with LCMV ARM or Cl13 exhibited high PD-L1 expression on plasmablasts at 9 Dpi. Infection of mice with LCMV ARM and with Cl13 has served as a powerful model to study immune responses during acute and chronic viral infections, respectively (2). In particular, LCMV Cl13, which produces chronic infection, induces higher and more sustained PD-1 upregulation in T cells, and PD-1/PD-L1 interaction significantly contributes to T cell suppression and viral persistence (45). Interestingly, LCMV infection also triggers polyclonal B cell activation (46, 47). Given that virus-specific CD4^+^ and CD8^+^ T cells from LCMV Cl13- (but not ARM-)-infected mice express high levels of PD-1 (45), it is tempting to speculate that PD-L1^hi^ plasmablasts may also contribute to suppressing antiviral T cells during chronic LCMV infection. It should be noted that, although PD-L1^hi^ plasmablasts were also induced after acute LCMV ARM infection, they are unlikely to exert a significant regulatory function in this setting, as virus-specific T cells from ARM-infected mice exhibit low levels of PD-1 expression (45).

It may be important to point out that plasmablasts may have more than one function. Plasmablasts produce cytokines (19, 48) and, therefore, can influence the response of other cell populations independent of PD-L1 expression. Plasmablasts secrete antibodies which could act directly on the parasite, controlling parasite replication (19). Therefore, we hypothesize that plasmablasts may act according to the context and the characteristics of the target cells.

In summary, our results revealed a new function of extrafollicular plasmablasts in the context of infections, which could provide a new target for the rational design of new therapeutic treatments aimed at enhancing protective T cell responses during Chagas disease, malaria and potentially other chronic infections.

## Methods

### Mice

C57BL/6, and B6.129S7-Ifngtm1Ts/J (IFNγKO) mice were initially obtained from The Jackson Laboratories (USA). These mice were housed and bred in the Animal Facility of the CIBICI-CONICET, FCQ-UNC.

Mice from the Australian National University Bioscience Services were maintained in specific pathogen-free conditions and had access to food and water ad libitum. The mice used in the experiments conducted at the John Curtin School of Medical Research (JCSMR) were:

HEL-IgM-BCR transgenic mice (MD4, knock in for a transgenic BCR specific for HEL, a *T. cruzi* non-related protein). As controls of the MD4 mice, we used their littermates whose genotype was negative for the knock in and therefore their B cells expressed a complete BCR repertoire.

SW_HEL_ CD45.1 mice, which carry a Vk10k light chain transgene and a knocked in VH10 Ig heavy chain in place of the JH segments of the endogenous IgH gene that encode a high-affinity antibody for HEL (hen egg lysozyme), were obtained from Robert Brink’s laboratory (Garvan Institute, Sydney, New South Wales, Australia). The BCR of all mature B cells recognize HEL.

Bcl6^fl/fl^ mice were mated to CD4-cre mice to generate Bcl6^fl/fl^ CD4^Cre^ mice (21). Mice expressing Cre lack Tfh, while Cre-negative T lymphocyte littermates will present a functional Bcl6 molecule and develop a normal Tfh response.

Mice experiments conducted at the CIBICI were approved by, and performed in accordance with the guidelines of, the Institutional Animal Care and Use Committee of the FCQ-UNC (Approval Number HCD 1525/14). Mice experiments conducted at JCSMR were approved by the Animal Experimentation Ethics Committee (Australian National University protocol number 2016/17). Male and female mice were age-matched (8-12 weeks-old) and housed with a 12-h light-dark cycle.

Mice experiments conducted at the Division of Molecular Biology, Department of Biological Sciences, University of California, San Diego, were handled in accordance with the requirements of the National Institutes of Health and the Institutional Animal Care and Use Guidelines of University of California, San Diego.

### Pathogens and Experimental Infections

*T. cruzi* infection: mice were infected intraperitoneally with 1 × 10^4^ trypomastigotes of *T. cruzi* Y-Br strain (49) diluted in a sterile solution of 1% glucose in PBS (23). Uninfected littermates were injected with 1% glucose in PBS and processed in parallel. Spleens were collected at different Dpi for immune response analysis. Bone marrow cells of *T. cruzi*-infected mice were isolated at 15 and 130 Dpi by flushing mice femurs and tibias with RPMI-1640.

*Plasmodium chabaudi* infection: mice were infected intravenously (iv) with 10000 parasites of *P. chabaudi* (strain AS) obtained from a mouse infected 8 days previously from a frozen stock. The spleens of *P chabaudi*-infected mice were obtained at 10 Dpi.

LCMV infection: mice were infected with 2 × 10^6^ plaque-forming units (pfu) of LCMV Arm (ARM) or LCMV clone 13 (Cl13) iv *via* tail vein. The Armstrong strain of LCMV causes acute infection in which the virus is cleared 8 days post infection, while the LCMV clone 13 causes chronic infection, with the virus persisting in the brain and kidneys for >3 mo (25).

Viruses were propagated on BHK cells and quantified by plaque assay performed on Vero cells (50). The spleens of LCMV-infected mice were obtained at 9 Dpi.

### Antigen Preparation

*T. cruzi* parasites (Y-Br strain) were cultured in NIH3T3 mouse fibroblasts and were collected as described (49). Trypomastigotes were washed twice in sterile PBS (37 °C) and were resuspended in 500 ul of PBS (4 °C) and sonicated in ice-cold water for 10 minutes. Aliquots were stored at -80 °C until use and thawed only once. Protein quantification was carried out after thawing in a Synergy HTX Multi-Mode Reader (Biotek).

### Immunizations

To evaluate PD-L1 expression on plasmablasts generated by TD-antigen immunization, two different schedules were performed: one group of C57BL/6 mice were immunized intravenously with 2 × 10^8^ SRBCs (Applied Biological Products Management, Australia) and the plasmablast phenotype was analyzed at 7 days post immunization (DPI). The other group of C57BL/6 mice were transferred, by iv injection, with splenocytes from CD45.1 SWHEL mice (Het/Het) containing 30.000 CD19^+^HEL^+^ cells simultaneously with 2x10^8^ Hel-conjugated SRBCs. The frequency of plasmablasts and plasmablast phenotypes was analyzed at 7 Dpi.

### Cell Preparation

Spleens were obtained and tissues were homogenized through a 0.70um cell strainer. Erythrocytes in splenic or bone marrow cell suspensions were lysed for 5 min in Tris–ammonium chloride buffer. Viable cell numbers were determined by trypan blue exclusion using a hemocytometer.

### Flow Cytometry

For surface staining, single-cell suspensions were washed first in ice-cold PBS and incubated for ten minutes with a Live/Dead Staining. Cells were washed in PBS and then incubated with fluorochrome-labeled Abs for 30 min at 4°C (surface staining). For intracellular cytokine staining, cells were cultured for 5 h with 50 ng/ml PMA (phorbol 12-myristate 13-acetate) (Sigma), 1,000 ng/ml ionomycin (Sigma) and Brefeldin A (eBioscience) or Monensin (eBioscience). Cells were fixed and permeabilized with BD Cytofix/Cytoperm and Perm/Wash (BD Biosciences) according to the manufacturer’s instructions. Data were collected on a BD FACSCanto II, BD LSR II, or BD Fortessa X20, and were analyzed using the FlowJo software (TreeStar). The specific cell populations and gating strategy in each case are described in **Figure S4**.

### Antibodies

The following anti-mouse antibodies were used for FACS: CD19-PE (6D5), CD19-BV605 (6D5), CD3-AlexaFluor700 (17A2), CD3-APCCY7 (17A2), CD4-APC-Cy7 (GK1.5), CD8-PerCP-Cy5.5 (53-6.7), CD8-FITC (5H10-1), Streptavidin BV605, PD-1-BV421 (29F.1A12), PDL-1-Pecy7 (10F.9G2), Bcl6-PE-Dazzle594 (7D1), ICOS-AlexaFluor488 (C398.4A), CD45.1- BV605 (A20), B220-APCCy7 (RA3-6B2), Live Dead Aqua 430 were from Biolegend; CD19-AlexaFluor700 (1D3), CD19-FITC (1D3), IgD-FITC (11-26c), CD8-PECy7 (53-6.7), TNF-APC (MP6-XT22), PD-L1-PE (MIH5) and were from eBioscience; CD138-BV605 (281-2), CD138-APC (281-2), CD138-PE (281-2), CD138- Biotin, CD38-BV421 (90/CD38), CXCR5-Biotin (2G8), IFN-γ-FITC (XMG1.2), Ki-67-AlexaFluor647 (B56), Blimp-1-PE-CF594 (5E7), and CD8-BUV805 (53-6.7) were from BD Biosciences.

The following anti-mouse antibodies were used for tissue immunofluorescence: B220-PE (RA3-6B2) and CD3 FITC anti-CD3 (145-2C11) from Biolegend and CD138-APC (281-2) from BD Biosciences.

HEL was conjugated with AlexaFluor647 with a protein labeling kit (Invitrogen).

### Immunofluorescence

For tissue immunofluorescence, spleens from infected WT mice were collected and frozen over liquid nitrogen. Frozen sections, 7 μm thick, were cut, fixed for 10 min in cold acetone, left to dry at 25 °C and stored at -80 °C until use. Slides were hydrated in TRIS buffer and blocked for 30 min at 25 °C with 10% normal mouse serum in TRIS buffer. After blockade, slides were incubated for 50 min at 25 °C with the different Abs diluted in TRIS Buffer. Slices were mounted with FluorSave (Merck Millipore). Images were collected with an Olympus microscope (FV1000) and those recorded in the far-red channel were pseudo-colored blue.

### Cell Culture

Splenic cells from infected mice were obtained at 14 Dpi and splenocytes were stained with fluorochrome-conjugated anti-CD19, anti-CD138 and anti-PD-L1. The elimination of plasmablasts, PD-L1^hi^, PD-L1^low^ and non-B cells from the splenocyte population was carried out by cell sorting in a FACS Aria II (BD Biosciences), using different gate strategies (see **Figure S3**).

Total splenocytes from infected mice or splenocytes depleted from plasmablasts, non-B cells, PD-L1^hi^ and PD-L1^low^ cells were incubated with AgTpY (5ug/mL) plus anti-PD-1, anti-PD-L1, anti-PD-L2 (5ug/mL) or control antibodies (5ug/mL). After 48 h of culture, TNF and IFNγ were quantified by ELISA in the culture supernatant according to the manufacturer’s instructions (eBioscience).

### Co-Cultures

CD4^+^ and CD8^+^ T cells were purified from a pool of the spleens of 2-3 non-infected C57BL/6 mice using EasySep Mouse CD4 or CD8 negative Selection Kit II (StemCell). CD19^+^ CD138^+^ plasmablasts were obtained from the spleens of *T. cruzi-*infected mice at 14 Dpi by cell-sorting using a FACS Aria IIb Cell Sorter (BD Biosciences). For sorting experiments, the spleens of at least 3 mice were pooled.

5x10^4^ CD4^+^ or CD8^+^ T cells were cultured alone or in combination with plasmablasts in a plate previously coated with anti-CD3 (1μg/mL, eBioscience) and anti-CD28 (0.25μg/mL, BD Biosciences) for 3 days in a 1:1 (T cell:plasmablast) or 1:2 ratio. When indicated, anti-PD-L1 or isotype control (both 5μg/mL) was added at the beginning of the cell culture. Next, cells were centrifuged and re-stimulated for 5 hours with PMA and ionomycin, and the frequency of TNF^+^ and IFNγ^+^ T cells was determined by flow cytometry.

### Statistics

Statistical significance through comparison of mean values was assessed by two-tailed Student’s t-test, one-way or two-way ANOVA, followed by the Bonferroni *post hoc* test using GraphPad software.

## Data Availability Statement

The original contributions presented in the study are included in the article/**Supplementary Material**. Further inquiries can be directed to the corresponding author.

## Ethics Statement

The animal study was reviewed and approved by Institutional animal Care and Use Committee Facultad de Ciencias Químicas - UNC.

## Author Contributions

MGS performed and designed most of the experiments, analyzed data and prepared figures and manuscript. FFV, CGB, LA, YG, MCR, and JTB collaborated with performing experiments. EIZ supervised design, analysis, and interpretation of the LCMV experiments. EW designed, performed, analyzed, and interpreted the LCMV experiments. IAC provided *P. chabaudi* parasites and contributed to the design of the *Plasmodium* experiments. IAC and YC performed the *Plasmodium* experiments. CLM and EVAR contributed to the study design and analysis. AG and CGV conceived, designed, and supervised the study and wrote the manuscript. All authors reviewed the manuscript before submission.

## Funding

This work was supported by grants from the Agencia Nacional de Promoción Científica y Técnica (PICT 2015-0645 and PICT 2018-01494), CONICET (PIP 201511220150100560CO) and R01AI116432 and R01AI110340 from NIH (USA). EVAR, CLM, and AG are Researchers from CONICET. MGS, FFV, LA, CB, YG, MR, and JTB, thank CONICET for the fellowship awarded. CGV was supported by Australian NH&MRC program, project, and fellowship grants. MGS was recipient of a Endeavour Scholarship.

## Conflict of Interest

The authors declare that the research was conducted in the absence of any commercial or financial relationships that could be construed as a potential conflict of interest.

## Publisher’s Note

All claims expressed in this article are solely those of the authors and do not necessarily represent those of their affiliated organizations, or those of the publisher, the editors and the reviewers. Any product that may be evaluated in this article, or claim that may be made by its manufacturer, is not guaranteed or endorsed by the publisher.
