# Supplementary figures and images for: Extrafollicular Plasmablasts Present in the Acute Phase of Infections Express High Levels of PD-L1 and Are Able to Limit T Cell Response
Source: Front Immunol. 2022 May 16;13:828734. doi: 10.3389/fimmu.2022.828734 (PMC9149371; doi:10.3389/fimmu.2022.828734)

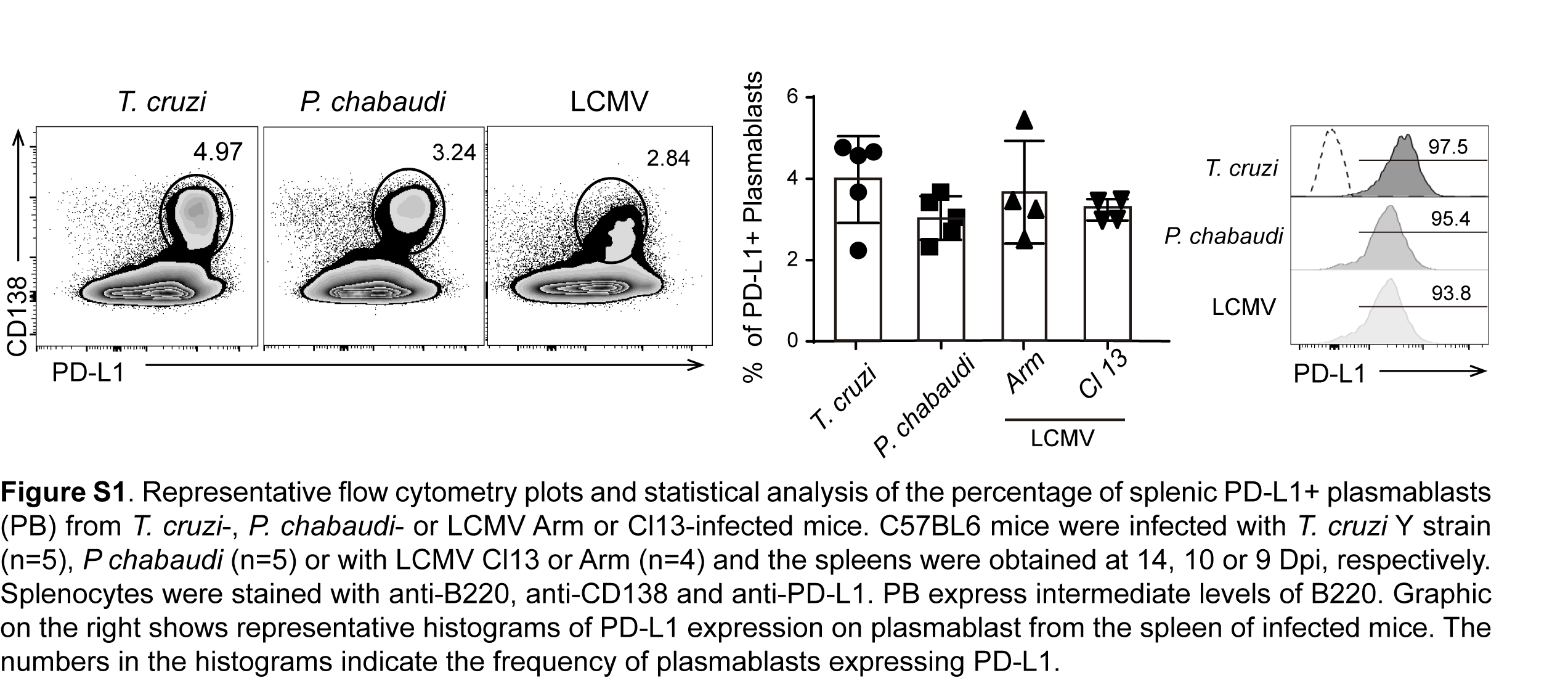

Supplement: Supplementary file 1 [file Image_1.tif]

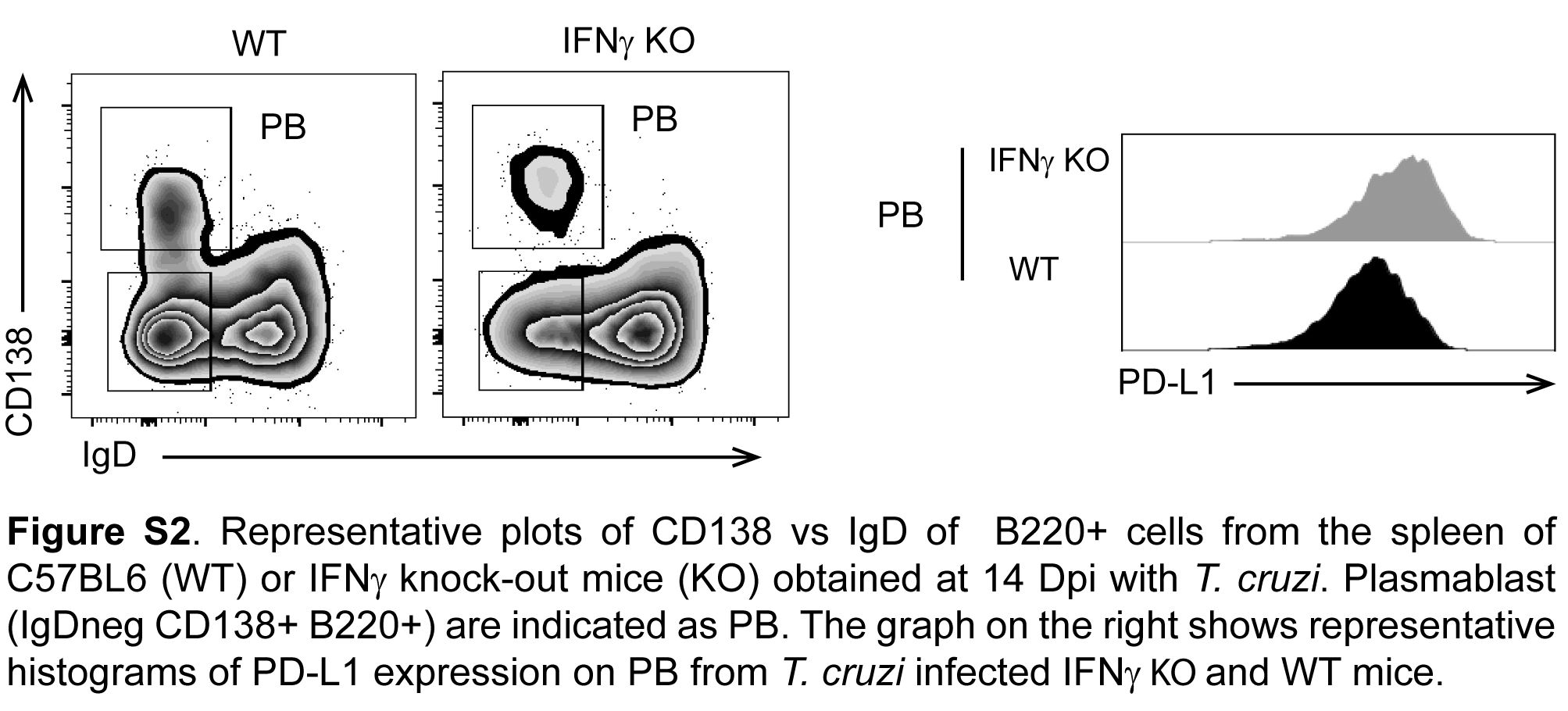

Supplement: Supplementary file 2 [file Image_2.tif]

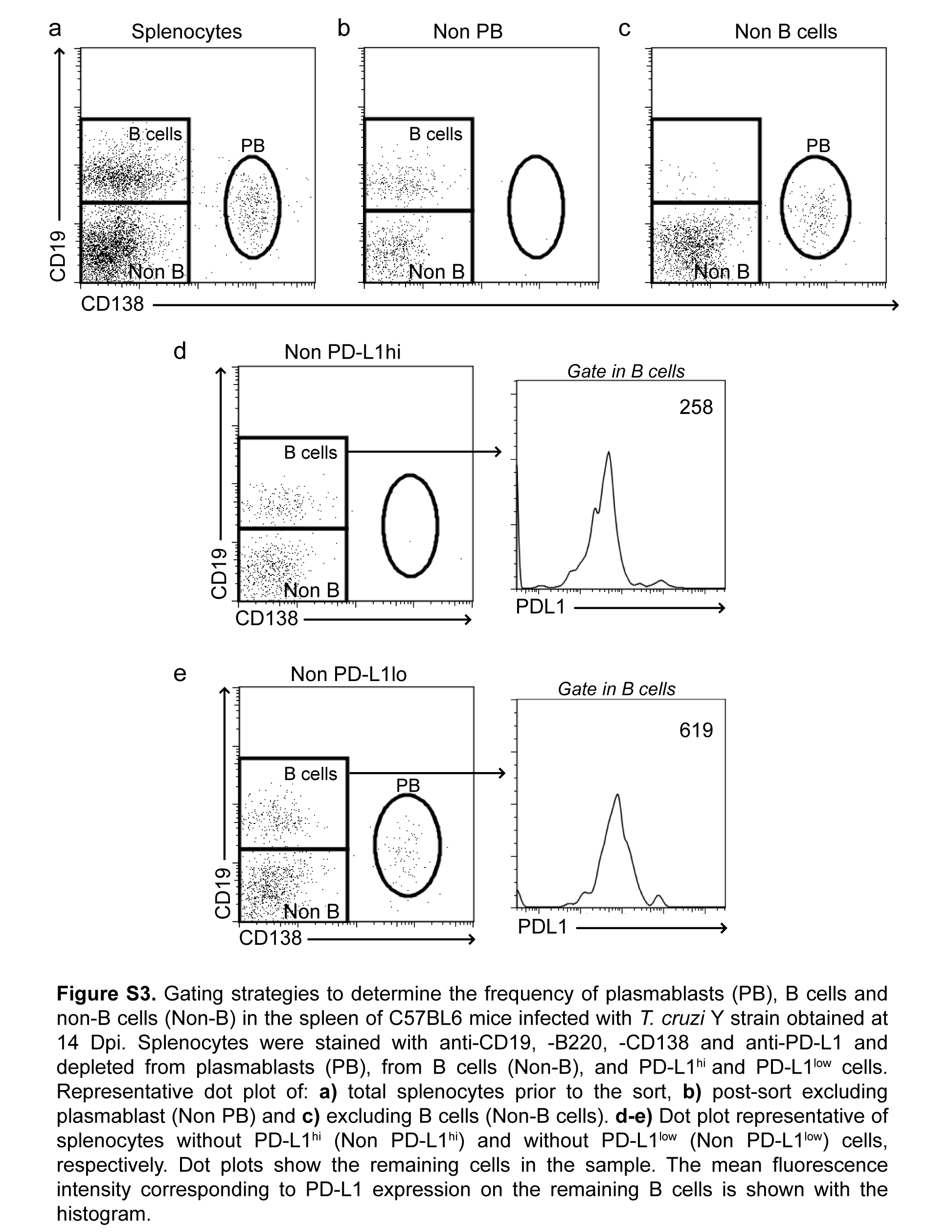

Supplement: Supplementary file 3 [file Image_3.tif]

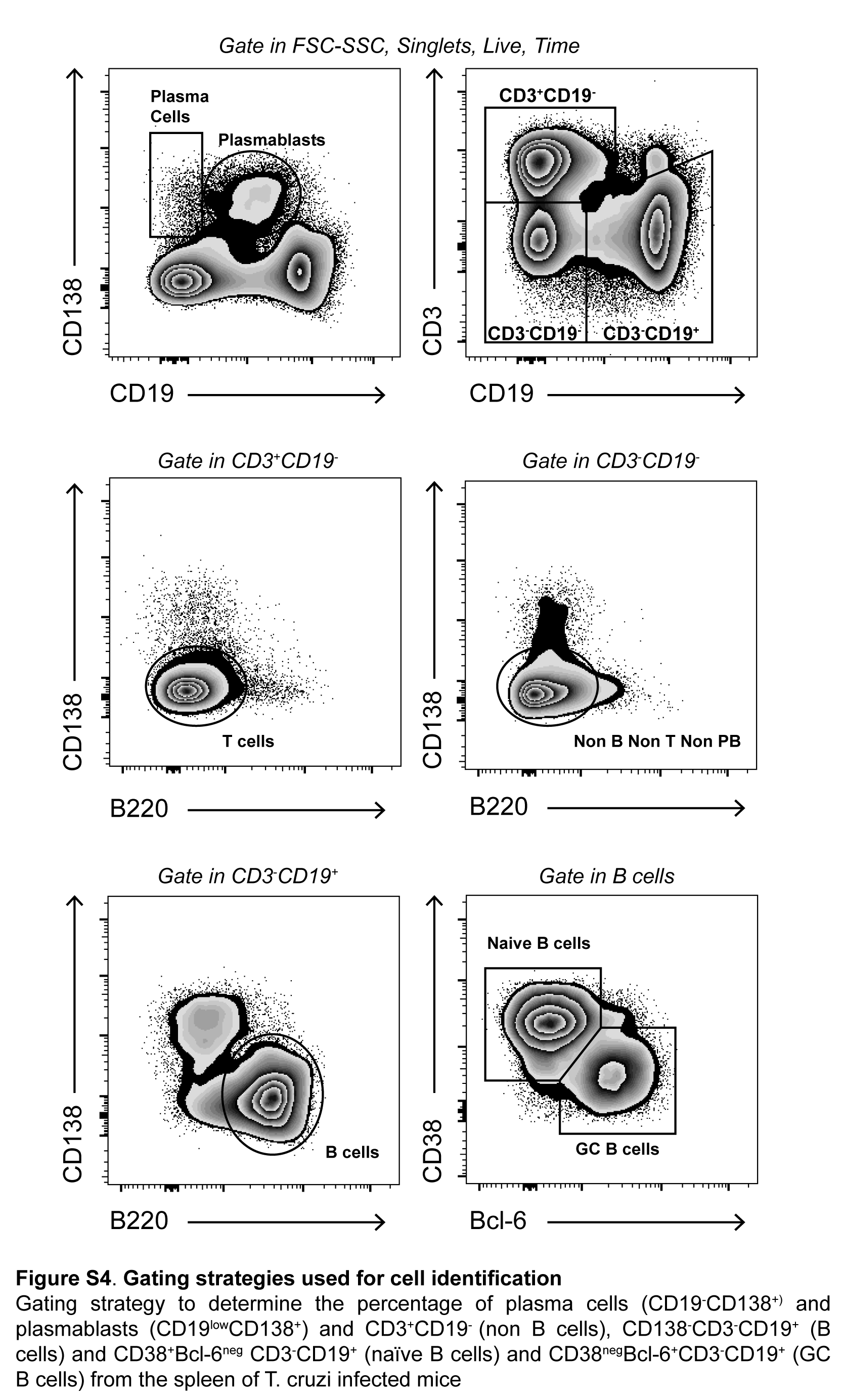

Supplement: Supplementary file 4 [file Image_4.tif]
